# The Landscape of Risk Communication Research: A Scientometric Analysis

**DOI:** 10.3390/ijerph17093255

**Published:** 2020-05-07

**Authors:** Floris Goerlandt, Jie Li, Genserik Reniers

**Affiliations:** 1 Department of Industrial Engineering, Dalhousie University, Halifax, NS B3H 4R2, Canada; floris.goerlandt@dal.ca; 2 Department of Safety Science and Engineering, School of Ocean Science and Engineering, Shanghai Maritime University, Shanghai 201306, China; lijie_jerry@126.com; 3 State Key Laboratory of Explosion Science and Technology, Beijing Institute of Technology, Beijing 100081, China; 4 Safety and Security Science, Faculty of Technology, Policy and Management, Delft University of Technology, 2628 BX Delft, The Netherlands; 5 Antwerp Research Group on Safety and Security (ARGoSS), Faculty of Applied Economics, University of Antwerp, 2000 Antwerp, Belgium; 6 Centre for Economics and Corporate Sustainability (CEDON), KU Leuven, 1000 Brussels, Belgium

**Keywords:** risk communication, scientometrics, bibliometrics, VOSviewer, CiteSpace

## Abstract

Risk communication is a significant research domain with practical importance in supporting societal risk governance and informed private decision making. In this article, a high-level analysis of the risk communication research domain is performed using scientometrics methods and visualization tools. Output trends and geographical patterns are identified, and patterns in scientific categories determined. A journal distribution analysis provides insights into dominant journals and the domain’s intellectual base. Thematic clusters and temporal evolution of focus topics are obtained using a terms analysis, and a co-citation analysis provides insights into the evolution of research fronts and key documents. The results indicate that the research volume grows exponentially, with by far most contributions originating from Western countries. The domain is highly interdisciplinary, rooted in psychology and social sciences, and branching mainly into medicine and environmental sciences. Narrative themes focus on risk communication in medical and societal risk governance contexts. The domain originated from public health and environmental concerns, with subsequent research fronts addressing risk communication concepts and models. Applied research fronts are associated with environmental hazards, public health, medical risks, nuclear power, and emergency response to various natural hazards. Based on the results, various avenues for future research are described.

## 1. Introduction

Risk communication is an essential aspect of risk management and governance. In the ISO 31000 standard for organizational risk management by the International Organization for Standardization [1], risk communication is part of the ‘communication and consultation’ activity of the risk management process, with the primary aims to promote awareness and understanding of risks. Although not without its critics [2,3] and notwithstanding ongoing work to define its conceptual basis [4], this standard is very influential across industrial domains as a platform for sharing best practices and as a catalyst for professionalization of risk management [5,6]. Through its provisions, the need for risk communication is highlighted, for instance, in supply chain risk [7], maritime oil spill preparedness and response [8], firefighting [9], and mining safety [10].

In the influential risk governance framework by the International Risk Governance Council [11], risk communication aims to ensure consideration of a plurality of values and interests in order to enable acceptance and social license of risk management strategies by societal actors. The IRGC’s framework has been applied in contexts such as food health and safety [12], drinking water quality [13], offshore oil [14], and autonomous vessels [15]. Within a risk governance context, several authors have described evolutions and trends in risk communication [16,17,18]. In general, these authors find that the focus in the early era was on explaining technical aspects of risk assessment, whereas more recent approaches focus on two-way communication with consideration of public concerns and risk perceptions, which is achieved through stakeholder involvement strategies.

Given the wide range of application domains where the importance of effective risk communication has been recognized, it is not surprising that risk communication has been an active research area. To provide summary insights into this increasingly extensive body of literature, several review articles have been published. Some of these focus on generic aspects of risk communication, such as its functions and associated problems [19], the assessment of effectiveness of communication interventions [20], the use of probabilistic information [21] or maps [22], or ethical aspects [23], while other reviews summarize the literature on the communication of specific risks such as vaccine-related risks [24], cancer screening [25], public health emergencies [26], and natural disasters [27].

While these reviews provide insights into specific approaches to risk communication, in some key aspects, or in research related to specific risks, there currently is no high-level analysis of the risk communication research domain. Scientometric analysis methods and associated visualization techniques enable obtaining insights into structural developments of a research domain, including temporal and geographical trends in outputs and focus topics, collaboration networks, scientific categories and thematic clusters, and co-citation networks and associated research fronts [28]. While narrative reviews are more suited to obtain focused insights into narrower issues [29], scientometric analyses have been developed to obtain knowledge about the high-level structure and dynamics of a research domain, using quantitative metrics and mathematical techniques [30,31,32]. Such high-level knowledge is primarily useful for researchers engaged in a given research domain to better understand its scope, nature, and development trends; its focus topics and themes; and key documents and authors. This is especially useful for early career researchers who are relatively new to the domain, but can also be helpful for experienced academics, for instance, in preparing lecture materials, or for journal editors to focus research attention on hot topics, e.g., by opening special issues on a specific theme. Several scientometric analyses have been published on risk, safety, health, and environment-related topics. These include an analysis of safety culture [33], road safety [34], resilient health care [35], sustainability and sustainable development [36], disaster risk management [37], slip and falls at worksites [38], electronic cigarettes [39], health and young people’s social networks [40], and process safety in China [41].

In light of this, the aim of this article is to present a scientometric analysis of the risk communication research domain. The specific research questions are as follows:

RQ1. What are the overall publication trends in terms of publication output?
RQ2. What geographic trends can be observed at a country level?
RQ3. What scientific categories are strongly represented?
RQ4. What journals are dominant knowledge carriers and what knowledge do these draw on?
RQ5. What are the dominant narrative topics, and what is their temporal evolution?
RQ6. What is the evolution in research clusters, associated research fronts, and key documents?

The remainder of this article is organized as follows. In Section 2, the document search strategy, data retrieval process, and resulting dataset are described, followed by a brief overview of the scientometric techniques and tools to answer the above research questions. The results and their interpretation are presented in Section 3. In Section 4, a discussion is given, contextualizing the work and providing directions for future research. Section 5 concludes.

## 2. Materials and Methods

There are four main steps in a typical scientometric analysis: formulating questions, data retrieval, application of suitable scientometric methods and tools, and interpretation of the results. The data retrieval strategy and resulting dataset is described in Section 2.1, and the scientometric methods and tools are briefly introduced in Section 2.2.

### 2.1. Data Retrieval Strategy and Resulting Dataset

The world’s largest and most comprehensive database of scientific publications, Web of Science Core Collections (WOSCC) was applied in this study to retrieve a high-quality dataset. Compared to other popular databases such as Scopus, SciFinder, or Google Scholar, WOSCC is the most comprehensive one across scientific disciplines, while also having a very high data quality [28]. The following search strategy was applied in the WOSCC database on 13 March 2020:

Title = “risk communication” AND
Document type = NOT (correction OR early access)

A title-based search strategy was applied in order to ensure that identified documents indeed focus on risk communication. A prior exploratory search based on title, abstract, and keywords led to a much larger dataset of over 5500 documents. Of these, many are however not directly relevant to obtaining insights into the risk communication research domain but instead mention risk communication more tangentially while focusing on risk perception, stakeholder participation, or other aspects of risk management or governance. With the applied title-based search process, all document types are retained in the resulting dataset, except articles presenting authors’ corrections to earlier publications and early access articles, i.e., articles which were not yet in final print at the time of the search. The timespan covered in the search ranges from 1900 until 2019 (inclusive).

The resulting dataset contained 1196 articles, which can be considered as the core scientific body of literature on risk communication. Table 1 contains some key descriptive information of this dataset, obtained through the R package Bibliometrix [42]. The results, which partially answer RQ1, show that risk communication research spans from 1985 to 2019, with 523 different journals contributing to the domain’s literature. In total, 3137 authors have (co-)authored at least one document, with only 296 authors having contributed a single-authored document. By far, most of the work is the result of multi-author collaboration, as can be seen from the high collaboration index of 3.39 and the average number of co-authors per document. The average number of citations per document is 14.82, which is relatively high. This indicates that risk communication research is quite impactful in the academic community.

### 2.2. Applied Scientometric Methods: Techniques and Tools

Various scientometrics methods were applied to answer the research questions listed in Section 1. Scientometric analysis involves the application of quantitative methods for detecting trends, patterns, and developments of a scientific research domain [43]. By visualizing quantitative metrics which represent informational aspects of the research domain, insights are obtained into its scope, contents, and development [44]. Table 2 contains an overview of the techniques and tools used to answer the research questions in this study. These are briefly described below.

Trends in research outputs (RQ1) are basic scientometric indicators, providing insights into the development of research activity over time. Apart from a simple count of publications per year, a regression analysis was performed to estimate the rate of change. Other basic trends of the publications in the research domain were determined by elementary summary statistics, using Bibliometrix software [42].

The geographic patterns (RQ2) were identified by counting the number of articles originating from the different countries/regions in the world. For each country-related subset of the data, additional metrics were calculated to provide insight in the temporal activity of different geographical areas and to assess the average impact of publications from the areas. Bibliometrix software was used for these basic calculations [42]. To identify collaboration networks between countries/regions in risk communication research, the visualization of similarities mapping technique was applied [45]. This technique quantitatively analyzes similarities between documents according to a chosen data object, in this case country/region of origin. The VOSviewer software determines citation networks in which the distance between nodes shows the level of closeness to each other and the node size represents the number of documents [50].

Insights into the scientific categories represented in risk communication research (RQ3) were obtained by mapping the journal categories on the global science map [46]. This map shows clusters of different scientific disciplines, providing a high-level visual overview of the complete scientific body of knowledge. Mapping the journal categories associated with the risk communication publications of the obtained dataset provides insights into what scientific domains actively contribute to the development of knowledge in this research area. The analysis and visualization were done with VOSviewer [45].

The information flow to and between journals as knowledge carriers in risk communication research (RQ4) was analyzed using the dual-map overlay [48]. This map shows the interconnections between over 10,000 journals, where these journals are grouped in regions representing publication and citation activities at the domain level [51]. The dual-map overlay enables insights into how specific domains of knowledge (citing articles) are influenced by other domains (cited literature), where the latter can be regarded as the intellectual base of the knowledge domain in focus [48].

The dominant narrative patterns in the risk communication domain (RQ5) were identified using the automatic term identification method [49] to extract terms or noun phrases from the bibliographic data about the documents in the dataset. In the present work, terms are extracted from title, abstract, and keywords. A data cleaning process was applied to combine similar terms in the resulting term list. VOSviewer [45] was applied to cluster the terms, to determine associated heat maps, and to obtain additional bibliometric indicators such as the average publication year and average impact of terms. This information provides insights into trending topics over time and helps to determine topics that are scientifically fruitful.

The evolution of research clusters, research fronts, and key documents (RQ6) was performed through a co-citation analysis using CiteSpace software [47]. Co-citation analysis was first proposed by Small [52] as a method to measure the relationship between two documents. Two documents are co-cited when they appear together in an article’s reference list. Resting on the premise that articles focusing on similar themes will cite partially the same articles, co-citation information in a set of documents provides high-level insights into the similarities between documents, from which research clusters can be identified. Recognizing that cited references can be considered indicative of the intellectual basis of a given area of research, the highly cited articles in these clusters can be considered key documents driving a domain of scientific work [53]. Furthermore, the articles citing most references from a given co-citation cluster are known as research fronts. In scientometrics research, these research fronts are considered to be the figureheads of a research cluster, providing insight in a subdomain of academic focus [54].

## 3. Results

In this section, the results of the various scientific analyses are shown and interpreted. Each subsection presents the analysis results to answer research questions RQ1 to RQ6.

### 3.1. Temporal Distribution

**Figure 1:**
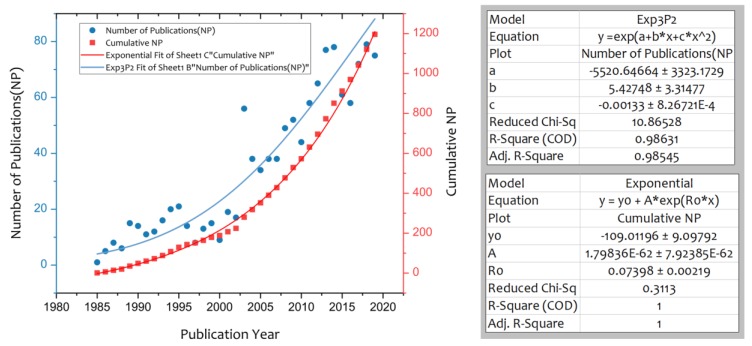
Yearly outputs of risk communication research.

The annual trend of publication activity in the risk communication research domain is shown in Figure 1. The first article was published in 1985, entitled “A Nonadvocate Model for Health Risk Communications”, authored by Petcovic and Johnson [55]. This indicates that risk communication research originates from a practical need to inform patients about health risks. The global trend of annual number of publications and the associated cumulative number shows an exponential increase. After a period with only a handful of publications annually at the initial stage of development of the research domain in the mid-1980s, a relatively steady stream of about 15 articles per year was published between about 1990 and 2000. From then onwards, the number of publications escalated quickly, with an increase to over 70 articles published annually after 2015. The research volume before 1990 amounts to 2.9% of the total, with the relative share of the period of 1990–1999 rising to 12.0%, further increasing to 29.3% in the period of 2000–2009, and finally reaching 55.8% in the period after 2010. This shows that risk communication research has experienced a rather dramatic increase in research productivity since its inception.

### 3.2. Geographical Distribution

**Figure 2:**
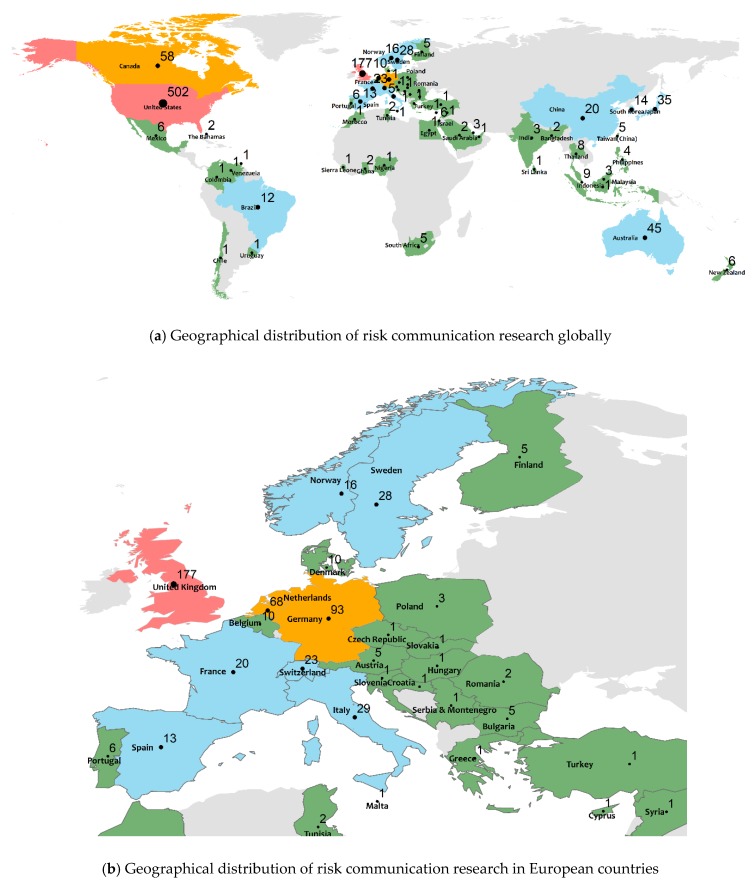
Geographical distribution of global risk communication research.

Figure 2 shows the geographic distribution of risk communication research globally. It is seen that, in total, 63 countries/regions have contributed to the 1196 documents comprising the dataset obtained in Section 2.1. The most productive countries, defined here as those with more than five publications, are listed in Table 3. For these countries, additional metrics including the average publication year and the average number of citations are determined as well.

It is seen that the vast majority of risk communication research originates from Western countries, with the United States of America (502 articles, 41.9%), the United Kingdom (177, 14.8%), Germany (93, 7.8%), the Netherlands (68, 5.7%), and Canada (58, 4.8%) comprising the top five most productive countries. The dominance of North America and Western Europe in research productivity is striking, while the research activity in Oceania, Asia, Eastern Europe, South America, and Africa is much lower. Australia and Japan are the only countries outside North America or Europe in the top 10. Within Europe, by far, most of the work originates from the United Kingdom, Germany, and the Netherlands, with Italy, Sweden, France, Norway, and Spain also contributing moderately. Eastern Europe is very poorly represented in risk communication research. In Asia, the research is most developed in the Far East, including Japan, the People’s Republic of China, and South Korea.

Despite the lower productivity in absolute terms, it is found that some countries in the list of Table 3, such as the People’s Republic of China and South Korea, have only relatively recently become active in this research domain. The top five most productive countries have been active for a much longer time, as seen from their comparatively low average year of publication. In terms of impact, the top highly productive countries also generally contribute the most impactful research. As is seen from the average number of citations, research originating from the USA, UK, Canada, and the Netherlands has attracted most citations on average, while work from some less productive countries including Switzerland, Israel, and Belgium also ranks relatively highly. The scientific impact of other countries is in general rather low, with average citation rates of around 5. This underscores the dominance of North America and Western Europe in the risk communication research domain.

**Figure 3:**
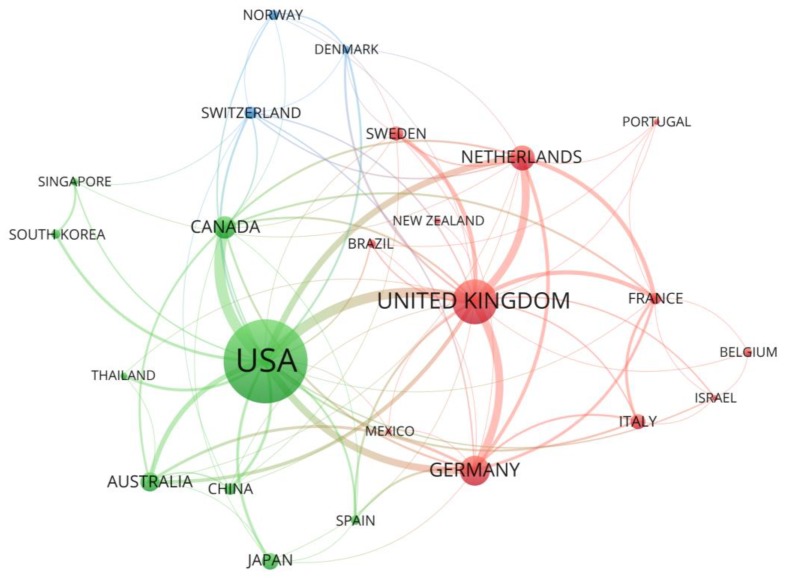
Countries/regions collaboration network in risk communication research (NP > 5).

The country collaboration network, shown in Figure 3, shows that the most active countries in North American and Western Europe, the United States of America and the United Kingdom, are also the ones with most international collaborations. Transatlantic collaboration is strongest between the USA and the UK, but Germany and the Netherlands also have such links. While the USA has the strongest academic links with Canada, Australia, Japan, China, and South Korea, the UK has stronger links with other European countries.

### 3.3. Scientific Categories

Each journal in the Web of Science Core Collection is classified according to different scientific categories. This categorization serves as a marker of the scientific disciplines and domains with which the journals are concerned. Aggregating these categorizations over the complete dataset obtained in Section 2.2 provides insights into how the risk communication research domain situates in the entire body of scientific knowledge.

**Figure 4:**
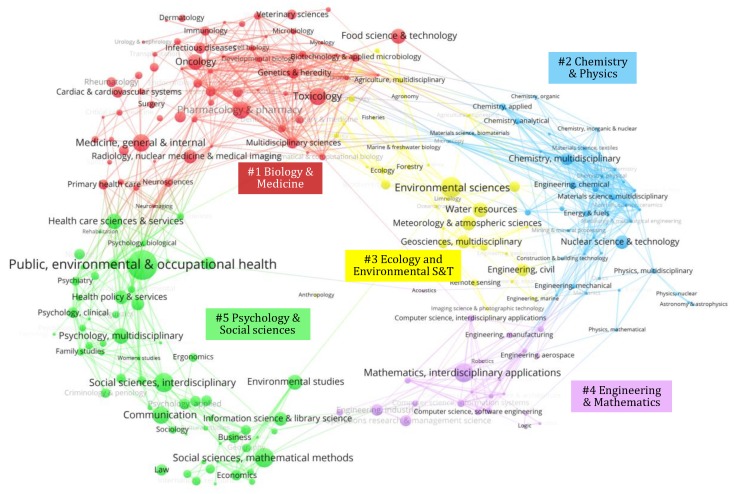
Scientific categories of risk communication research on global science map.

The distribution of scientific categories associated with risk communication is shown on the global science map [56] using the VOSviewer software [45]. The results are shown in Figure 4, where the global scientific categories are grouped in five clusters. These are #1 ‘*Biology and Medicine*’, #2 ‘*Chemistry and Physics*’, #3 ‘*Ecology and Environmental Science and Technology*’, #4 ‘*Engineering and Mathematics*’, and #5 ‘*Psychology and Social Sciences*’.

Table 4 provides an overview of the most frequently occurring scientific categories in risk communication research, here defined as categories in which at least 20 articles are classified. Furthermore, the average publication year and average number of citations of these categories are shown, providing insight in the temporal evolution of and the scientific impact associated with these categories. The table also indicates which cluster of Figure 4 the scientific category is located in, for easier interpretation of the figure.

The results indicate that risk communication research is primarily located in the ‘*Psychology and Social Sciences*’ scientific domain (cluster #5). Within that cluster, the scientific categories ‘*Public, Environmental, and Occupational Health*’ (362 articles, 30.3% of the total dataset), ‘*Social Sciences, Mathematical Methods*’ (89, 7.4%), ‘*Social Sciences, Interdisciplinary*’ (86, 7.2%), ‘*Communication*’ (67, 5.6%), and ‘*Psychology, Multidisciplinary*’ (42, 3.5%) are the most actively contributing. The second most prevalent scientific domain is ‘*Biology and Medicine*’ (cluster #1), in which the scientific categories ‘*Medicine, General and Internal*’ (67, 5.6%), ‘*Toxicology*’ (65, 5.4%), ‘*Pharmacology and Pharmacy*’ (64, 5.3%), ‘*Oncology*’ (42, 3.5%), and ‘*Food Science and Technology*’ (39, 3.3%) are the highest contributors. The third most significantly contributing scientific domain is ‘*Ecology and Environmental Science and Technology*’ (cluster #3). Here, the scientific categories ‘*Environmental Sciences*’ (105, 8.8%), ‘*Water Resources*’ (41, 3.4%), ‘*Meteorology and Atmospheric Sciences*’ (34, 2.8%), and ‘*Geosciences, Multidisciplinary*’ (24, 2.0%) are highly contributing scientific categories. The scientific domains ‘*Engineering and Mathematics*’ (cluster #4) and *‘Chemistry and Physics’* (cluster #2) are contribute significantly less to the risk communication research domain, with only ‘*Mathematics, Interdisciplinary Application*’ (88, 7.4%), ‘*Nuclear Science and Technology*’ (28, 2.3%), and ‘*Engineering, Civil*’ (21, 1.8%) being highly contributing scientific categories.

Apart from highlighting the main contributing scientific categories, the visualization of risk communication research on the global science map in Figure 4 also indicates that this research domain is highly interdisciplinary. While the research domain appears to have a very application-oriented focus, especially on health and environmental risks, its scientific foundations lie in social sciences. Furthermore, mathematical methods and their interdisciplinary application in social sciences also are an important aspect in the research domain. While there are some generic scientific categories of the social sciences represented, e.g., ‘*Social Sciences, Interdisciplinary*’ and ‘*Psychology, Multidisciplinary*’, the only significantly contributing specific communications-oriented social science categories with specific relevance to the domain’s conceptual basis are ‘*Communication*’ and ‘*Information Science and Library Science*’. This shows that most work in the risk communication domain originates from practical needs in specific risk management and governance contexts, rather than as a subdiscipline from communications research.

To further support the finding that risk communication is highly interdisciplinary, the Stirling-Rao diversity index is calculated. This metric measures the aggregate distance between connected scientific categories, giving more weight to connected article pairs associated with more distant categories [57]. For the risk communication research domain, the global diversity index is 0.803, which is a very high score. This indicates that there is a high diversity in scientific categories concerned with this domain, and that these collectively contribute to the knowledge production.

Focusing on Table 4, the average year in which articles in a category are published shows that the oldest categories are ‘*Social Sciences, Mathematical Methods*’ and ‘*Mathematics, Interdisciplinary Applications*’, which are among the most active categories overall. Most application-oriented categories have an average publication year around 2010, with some variation. Categories in which the contributions appear significantly earlier (average before 2008) are ‘*Engineering, Civil*’, ‘*Nuclear Science and Technology*’, ‘*Public, Environmental, and Occupational Health*’ and ‘*Environmental Sciences*’. More recently emerging categories (average after 2012) include ‘*Meteorology and Atmospheric Sciences*’ and ‘*Geosciences, Multidisciplinary*’. In terms of research impact, it is found that several categories from cluster #5 ‘*Psychology and Social Sciences*’ are highly impactful, including ‘*Information Science and Library Science*’, ‘*Social Sciences, Mathematical Methods*’, ‘*Health Policy and Services*’, and ‘*Health Care Sciences and Services*’. In other science clusters, impactful categories are ‘*Mathematics, Interdisciplinary Applications*’ (cluster #4), ‘*Medical Informatics*’ and ‘*Medicine, General and Internal*’ (cluster #1). Remarkably, highly productive application-focused categories in other scientific clusters are much less academically impactful, with even categories which became active comparatively early, such as ‘*Environmental Sciences*’ and ‘*Water Resources*’ (cluster #3), ‘*Engineering, Civil*’ (cluster #4), and ‘*Nuclear Science and Technology*’ (cluster #2) receiving few citations on average. This shows that, in general, medicine- and health-related risk communication work is more impactful. Nevertheless, the above-identified recently emerging categories ‘*Meteorology and Atmospheric Sciences*’ and ‘*Geosciences, Multidisciplinary*’ (cluster #3) also have a comparatively high average number of citations and hence academic impact, given their relatively short time to attract citations.

### 3.4. Journals’ Distribution and Intellectual Base

**Figure 5:**
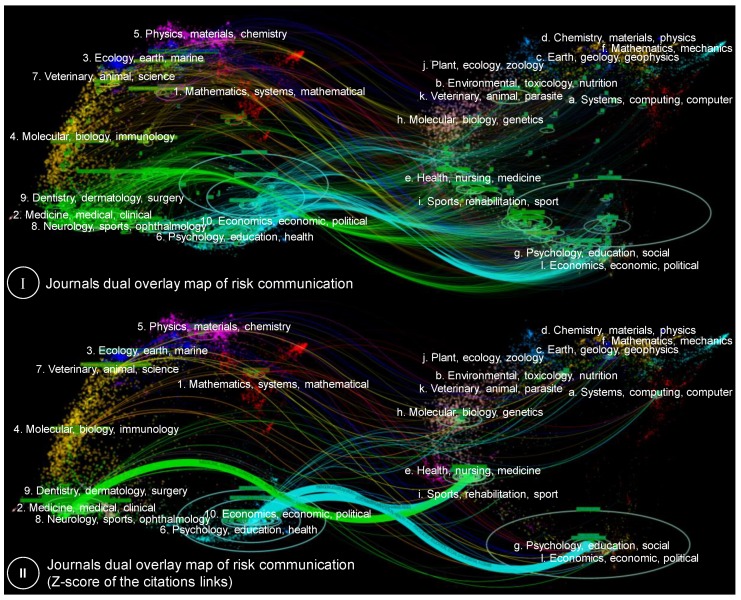
Dual-map overlay of risk communication papers on the global science map, on the basis of journals.

A dual-map overlay analysis is applied to identify highly productive and highly cited journals in the risk communication research domain and to trace their intellectual basis. The results are shown in Figure 5 and Table 5.

The dual-map overlay analysis is performed using CiteSpace [47] and the journal-based dual-map overlay created by Carley and his colleagues [46]. It shows the journals of a specific dataset (here the risk communication dataset of Section 2.2) on the global science map of journals. The analysis then traces the cited journals in the reference list of those journals, puts those on another journal overlay map, and links both maps. To facilitate the interpretation, labeled ovals are used to indicate clusters of highly active citing and cited journals. The size of the ovals is proportionate to the number of publications for the citing journals on the left and to the number of citations received from the risk communication articles by a journal on the right. Thus, on the left-hand side of the upper part of Figure 5, the distribution of risk communication journals on the global science map is shown, whereas the right-hand side shows the distribution of cited journals. The bottom part of Figure 5 further condensed the information by concentrating lines between citing and cited journal clusters. This is done by adjusting the width of the lines proportional to the frequency of citation, making use of the so-called z-score of the citation links [51].

The upper part of Figure 5 shows that risk communication articles are mainly published in ‘*Psychology, Education, Health*’ and ‘*Medicine, Medical, Clinical*’ journal groups. The cited journals, which can be considered to constitute the intellectual basis of the research domain, are primarily clustered in the ‘*Health, Nursing, Medicine*’ and ‘*Psychology, Education, Social*’ journal groups. The lower part of Figure 5 shows the main journal groups and their connections, where the line widths are scaled using the z-score. It is seen that journals from the ‘*Psychology, Education, Health*’ journal groups in risk communication research mainly have cited journals from the ‘*Health, Nursing, Medicine*’ and ‘*Psychology, Education, Social’* groups. The citing journals from ‘*Medicine, Medical, Clinical*’ have predominantly cited journals from the ‘*Health, Nursing, Medicine*’ group. This is also reflected in the results of the calculated z-scores for the citation trends at the domain level, as shown in Table 6.

It is also seen that nearly all citing journal groups cite journals from the ‘*Psychology, Education, Social’* journal group, while furthermore relying on a relatively small group of journal domains, mostly health- and environment-related. This implies that, despite the high level of interdisciplinarity as found in Section 3.3, the intellectual basis of risk communication research remains relatively focused within specific scientific subdomains. Articles furthermore appear to often cite articles from their own journal group.

Table 5 shows the top 10 highly productive citing journals of the risk communication research domain, as well as the journals with the highest number of citations. It is seen that *Risk Analysis* and *Journal of Risk Research* are by far the most productive journals, followed at a distance by medical- and health-related journals such as *Drug Safety* and *Journal of Health Communication*. For the cited journals, it is found that by far most references are received by *Risk Analysis*, with *British Medical Journal*, *Medical Decision Making*, *Journal of Risk Research*, and *Science*.

### 3.5. Terms Analysis: Narrative Patterns

**Figure 6:**
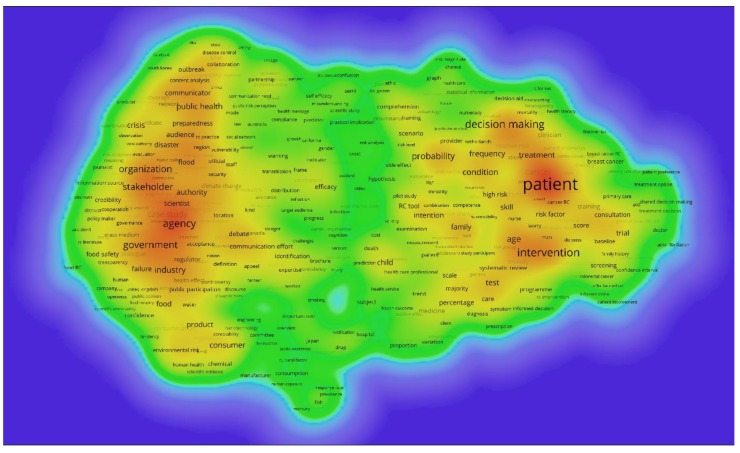
Term density map of risk communication research, all documents (458 terms included).

**Figure 7:**
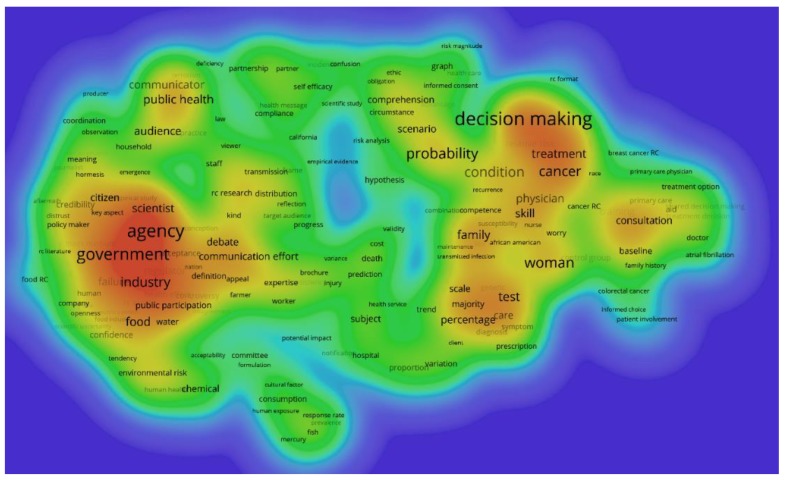
Term density map of risk communication research, average publication year before 2010, (227 terms included).

**Figure 8:**
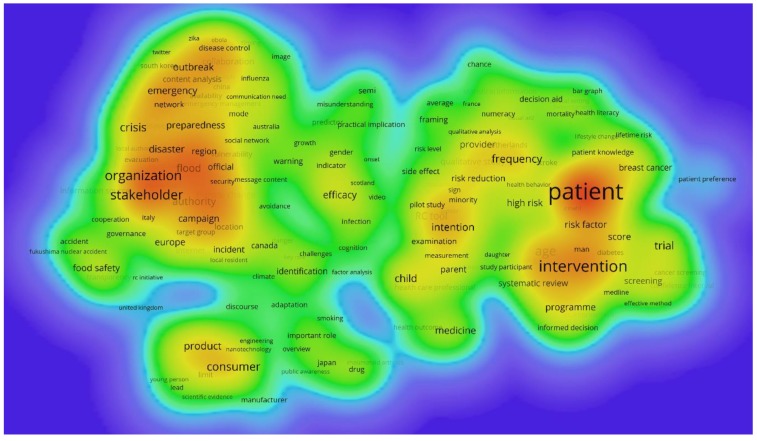
Term density map of risk communication research, average publication year after 2010, (231 terms included).

The automatic term identification method in the VOSviewer software [45,49] is applied to extract terms and noun phrases related to the risk communication dataset of Section 2.1. In the present work, these are extracted from the title, abstract, and keywords. Only terms which appeared at least five times are retained for further analysis, with similar terms are merged to increase clarity in and focus of the results, as is commonly recommended in scientometric analyses [28]. In total, 458 terms are retained, which are clustered using VOSviewer and subsequently transformed in heat maps to identify concentrations of higher activity. Figure 6 shows the dominant narrative patterns of the entire dataset, indicating the existence of two large clusters. Table 7 lists the terms analysis results for these two clusters, along with additional information such as the number of occurrences, the average publication year in which the terms appeared, and the average citations received. Additionally, Figure 7 and Figure 8 show a term density map of the term clusters by average year of publication of the terms, which highlights the temporal evolution of the clusters.

In the left cluster in Figure 6 (Cluster A in Table 7), the main terms are ‘*agency*’, ‘*government*’, ‘*stakeholder*’, ‘*organization*’, and ‘*case study*’, whereas in the right cluster (Cluster B in Table 7), the most frequently occurring terms are ‘*patient*’, ‘*intervention*’, ‘*decision making*’, ‘*probability*’, and ‘*woman*’. On a high level, this indicates that the risk communication domain contains two major domains of work. On the one hand, there is a role for risk communication in societal risk governance, where governmental agencies interact with stakeholders from industry, the public, and academics in regard to societal risks, as in the IRGC risk governance framework [11] mentioned in the introduction. On the other hand, there is an important role for risk communication on a more personal level in medical contexts, where medical practitioners interact with patients about treatments of specific medical conditions, as in the guidance by the Risk Communication Institute [58]. The most frequently occurring keywords here are ‘*patient*’, ‘*intervention*’, ‘*decision making*’, ‘*probability*’, and ‘*woman*’.

Table 7 and Figure 6 show that risk issues around ‘*public health*’, ‘*food*’, ‘*floods*’, ‘*disasters*’, (disease) ‘*outbreak*’, and ‘*emergency*’ are important topics in cluster A (societal risk governance). Methodological and conceptual aspects of risk communication in societal risk governance such as ‘*debate*’, ‘*public perception*’, ‘*dialogue*’, ‘*social medium*’, and ‘*credibility*’ are important in this narrative. From Figure 7 and Figure 8 and Table 7, it is found that earlier narratives were more strongly focused on government agencies, industry, scientists, and public participation. Topics included public health, environmental risks, and food. Dominant narratives after 2010 became stakeholders and organizations, with more attention to emergencies, crises, disasters, preparedness, outbreaks and disease control, and consumer products. Academically impactful methodological narratives in Cluster A revolve around communicators, communication efforts and efficacy, audience, public perception, and public participation. Impactful topic-focused narratives concern disaster, crisis, emergency, and flood.

In Cluster B (medical risk communication), important narratives revolve around risk issues such as ‘*treatment*’, ‘*age*’, ‘*family*’, ‘*cancer*’, ‘*diagnosis*’, ‘*medicine*’, and ‘*screening*’. Methodological and conceptual aspects of medical risk communication include ‘*probability*’, ‘*scale*’, ‘*scenario*’, ‘*skill*’, ‘*decision making*’, ‘*test*’, and ‘*patient knowledge*’. Inspecting Figure 7 and Figure 8 and Table 7 shows that narratives around decision making, probability, treatment, cancer, family, woman, and consultation were dominant before 2010. After 2010, narratives focused more on patients, intervention, risk factors, age, and intentions. Academically impactful narratives in Cluster B involve skill, relative risk, scale, decision making, subject, systematic review, tests, and frequency.

Overall, the results show that some narratives are rather robust in the risk communication research domain, with a continued focus on patient-, treatment-, and risk-related information in Cluster B and a continued attention to societal health risks. The results also indicate that risk communication in emergency and disaster contexts has become a topic of academic interest more recently.

### 3.6. Cited References—Research Fronts

CiteSpace [47] is applied in this section to perform a co-citation analysis of the risk communication dataset of Section 2.1 in order to determine research clusters based on co-citation information. Co-occurrence of certain references in a set of articles within a research domain is a commonly used technique in scientometric research to identify clusters [28]. Highly cited references within these clusters can be understood as the intellectual basis of the subdomains and represent key knowledge carriers for the development of the research domain. Articles citing the largest number of references from a cluster are known as ‘research fronts’. These can be seen as spearheading contributions leading the development of the research domain, and together they provide insight in the overall evolution of the research domain in terms of focus topics [38,51].

**Figure 9:**
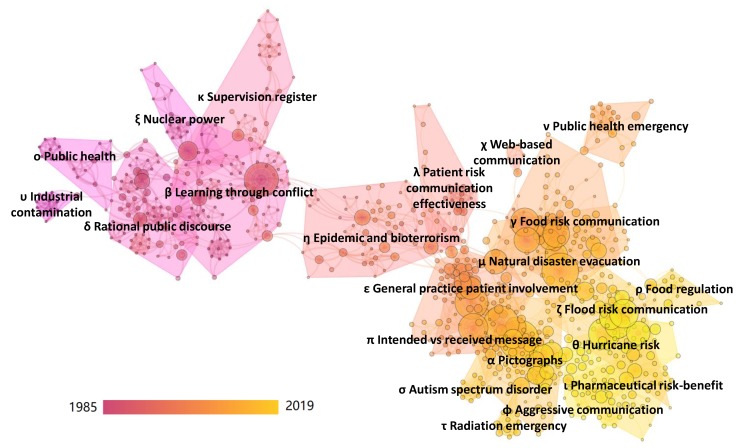
Clusters of co-citation network of risk communication research.

In order to obtain a clear structure of the results, the co-citation analysis is here performed for the entire timespan of the dataset (1985–2019), using a time slice length of one year, an eight year look-back period of considering cited references, and a minimum of two citations per period. The resulting co-citation network has 1157 nodes and 3924 co-citation links. The largest connected component of this co-citation network is shown in Figure 9 to show the most important parts of the structure and the intellectual basis of the research clusters. The labels of the clusters determined by CiteSpace are extracted from the title of the citing publications, based on the log-likelihood ratio (LLR) method. In the figure, the node sizes are proportional to the number of citations of a publication, while the colors of the links between articles indicates the year when two documents were first cited together. The color shade of the clusters indicates the average publication year of the references. The main analysis results of the co-citation analysis for the largest network of connected clusters is shown in Table 8. This table shows the name of the research cluster, the number of references included in the cluster, the associated article representing the research front, the average year of publication of the cited references, and the silhouette value. The silhouette value of a cluster ranges from −1 to 1 and indicates the uncertainty which needs to be considered when interpreting the nature of the cluster. A value of 1 represents a perfect separation from other clusters [59].

**Figure 10:**
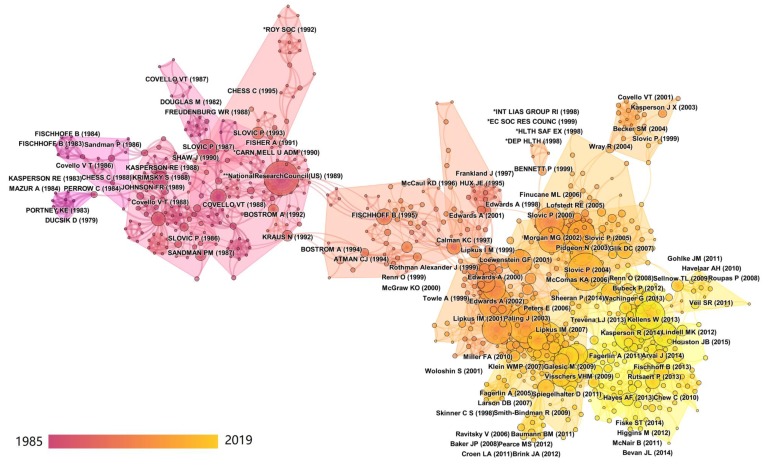
Top five highly cited references in each co-citation network cluster.

In Figure 10, the five most highly cited references in each research cluster are shown. As explained above, these can be considered as the intellectual base of each subdomain of risk communication research. Table 9 provides additional information of the top five highly cited references in the largest co-citation clusters, defined here as clusters with a minimum of 50 articles, as shown as well in Table 8. Only references with a minimum of five citations are retained.

The landscape and time evolution of the clusters shows that the earliest research fronts of risk communication research focus on ‘*υ Industrial Contamination*’ and ‘*σ Public Health*’, with 1982 and 1986 being the average publication years of the cited references, respectively. This indicates that risk communication research arose from a practical need to inform the public about health and environmental risks. Thereafter, there were several research clusters which focused on better understanding risk communication as an activity in itself, which can be considered as a type of fundamental risk research [67]. These include ‘*δ Rational Public Discourse*’ (average publication year of cited references: 1988), ‘*β Learning through Conflict*’ (1989), ‘*π Intended* vs. *Received Message*’ (2000), and ‘*φ Aggressive Risk Communication*’ (2012). Nevertheless, the bulk of the risk communication research clusters remained focused on specific risk issues throughout the evolution of the research domain, in line with societal concerns or contemporary focus topics in medical research. Examples of such research clusters associated with the societal risk governance cluster (Cluster A of Section 3.5) include ‘*ξ Nuclear Power*’ (1986), ‘*η Epidemic and Bioterrorism*’ (1996), ‘*μ Natural Disaster Evacuation*’ (2005), ‘*ζ Flood Risk Communication*’ (2009), and ‘*θ Hurricane Risk*’ (2013). Examples of research clusters associated with medical risk communication research (Cluster B of Section 3.5) include ‘*κ Supervision Register*’ (1992), ‘*λ Patient Risk Communication Effectiveness*’ (1997), ‘*ε General Practice Patient Involvement*’ (2001), and ‘*ι Pharmaceutical Risk–Benefit*’ (2012).

Referring to Table 8 and Table 9, the largest cluster spans 84 articles with a silhouette value of 0.769, indicating a relatively large overlap with other clusters. It is labeled ‘*α Pictographs*’ based on LLR analysis. The research front is [60], which focuses on the use of pictographs for communicating medical screening information to persons with higher and lower numeracy skills. This cluster is associated with Cluster B (medical risk communication) of Section 3.5, draws on ‘*Health, Nursing, and Medicine*’ and ‘*Psychology, Education, Social*’ journals of the global science map of Section 3.4, and involves scientific categories in the clusters ‘*#1 Biology and Medicine*’ and ‘*#5 Psychology and Social Sciences*’ on the global science map of Section 3.3. The most highly cited reference in this cluster is [68], which focuses on best practices on conveying health risks using numeric, verbal, and visual formats. Other highly cited references include [58,69,70,71], which focus on patient understanding of risks, numeracy, and the relation to decision making. The cluster also contains a review on the use of probability information in risk communication [21,71].

The second largest cluster is labeled ‘*β Learning through Conflict*’. It includes 78 references with a silhouette value of 0.931, indicating that it is well separated from other clusters. Its research front is [61], which focuses on the role of conflict in risk communication, as a means of learning in contexts where controversy exists between stakeholders. This cluster is associated with Cluster A (societal risk governance) of Section 3.5, draws on mainly on journals from ‘*Psychology, Education, Social*’ journals on the global science map of Section 3.4, and is based on the scientific category ‘*#5 Psychology and Social Sciences*’ on the global science map of Section 3.3. The reference with highest number of citations is [72], a book on risk communication aimed at decision-makers in government and industry, highlighting both the importance of procedure and content of risk messages. Other significant references are [73], a manual for industrial managers outlining a number of key rules for communicating with the public; [74], which outlines a mental model of how lay people respond to environmental hazards; and [75], which studies differences in lay and expert judgments of toxicological risks.

The third largest cluster is labeled ‘*γ Food Risk Communication*’, which includes 75 references with an average publication year of 2003 and a silhouette value of 0.867, indicating a reasonable separation of other research clusters. Its research front is [62], which describes the history of risk communication, summarizes theoretical avenues, and provides research directions in food-related risks. It highlights media amplification, public trust, and communication of uncertainty as essential ingredients. This cluster is associated with Cluster A (societal risk governance) of Section 3.5, draws on mainly on journals from ‘*Psychology, Education, Social*’ journals on the global science map of Section 3.4, and is based on the scientific categories ‘*#5 Psychology and Social Sciences*’ and ‘*#1 Biology and Medicine*’ on the global science map of Section 3.3. The reference with highest number of citations in this cluster is [76], a highly influential book in risk research, focusing on the conceptual and methodological basis of risk perception and its implications. Other impactful references include [77], a book outlining the social amplification of risk framework, and [78], a book describing theory and applications of a mental models approach to risk communication. The last two highly impactful references in this cluster are [18], a review article describing the evolution of some major developments in risk communication in the period 1996–2005, and [79], a book outlining four risk management strategies (political regulatory process, public deliberation, the technocratic/scientific perspective, and strictly economics-based risk management) and risk management case studies in Germany, the USA, and Sweden.

The fourth largest cluster is ‘*δ Rational Public Discourse*’, which includes 69 references with an average publication year of 1988. Its silhouette value is 0.898, indicating a high degree of separation of other co-citation clusters. The research front of this cluster is [63], which discusses a communication process between stakeholders with conflicting interests from the viewpoint of message recognition, inducing attitude and behavior changes, and conflict resolution. This cluster is associated with Cluster A (societal risk governance) of Section 3.5, is based on knowledge from journals related to ‘*Psychology, Education, Social*’ on the global science map of Section 3.4, and is strongly rooted in the scientific category ‘*#5 Psychology and Social Sciences*’ on the global science map of Section 3.3. The reference with the highest number of citations in this cluster is [80], an influential book on risk communication, introducing it as a technical and cultural phenomenon. Another influential reference is [81], a book outlining seven cardinal rules for effective risk communication in environmental risk management. The final two highly influential references in this cluster are [82], which introduces the social amplification of risk framework, and [83], which presents results of a study on risk communication in response to public concerns about geological radon health hazards.

The fifth largest co-citation cluster, spanning 61 references with an average publication year of 2001, is labeled ‘*ε General Practice Patient Involvement*’. It has a silhouette value of 0.835, indicating a relatively large overlap with other clusters. The research front of this cluster is [64], which presents results of a study on the use of risk communication for shared decision making in general practice. It is associated with Cluster B (medical risk communication) of Section 3.5 and involves knowledge from journals related to ‘*Health, Nursing, Medicine*’ and ‘*Psychology, Education, Social*’ on the global science map of Section 3.4. It involves interdisciplinary scientific categories, bridging the scientific domains ‘*#1 Biology and Medicine*’ and ‘*#5 Psychology and Social Sciences*’ on the global science map of Section 3.3. The most impactful reference in this cluster is [84], which studies how numerical information can be visually represented to support dialogue and risk communication in medicine. Other highly impactful references include [20,85,86,87,88], which concern various aspects of the visual communication of medical-related risks and the impacts on effectiveness of patient decision making. The references [89,90] address case studies of representation of risk information related to violence and cancer, whereas [91,92] address patient participation and teaching and learning in shared decision making.

The sixth largest research cluster spans 56 references with an average publication year of 2009 and is labeled ‘*ζ Flood Risk Communication*’. With a silhouette value of 0.852, it has a relatively large overlap with other clusters. The research front of this cluster is [65], which describes a best practices model for risk communication and management in environmental hazards related to floods. This cluster is associated with Cluster A (societal risk governance) of Section 3.5, relies on journals focusing on ‘*Psychology, Education, Social*’ on the global science map of Section 3.4, and bridges the scientific domains ‘*#5 Psychology and Social Sciences*’ and ‘*#3 Ecology and Environmental Science and Technology*’ on the global science map of Section 3.3. The highest cited reference in this cluster is [93], which builds on an extensive body of risk communication literature to address four questions about risk communication, including how to communicate uncertainty, how declining trust can be handled, and what the lessons learned from earlier work can be used to define new principles for risk communication. Other influential work in this cluster includes [94], which addresses risk perception and communication in natural hazards; [95,96], which review perceptions on flood risks and associated flood mitigation behavior; and [97], a book outlining an earlier version of the risk governance framework by the International Risk Governance Council [11].

Finally, the seventh largest co-citation cluster is labeled ‘*η Epidemic and Bioterrorism*’. With 55 references and an average publication year of 1996, it is the last cluster with more than 50 references included. It has a silhouette value of 0.934, indicating a good separation from other co-citation clusters. Its research front is [66], which is a highly impactful article describing risk perceptions and communication strategies for release of a biohazard pathogen in an urban setting. This cluster is associated with Cluster A (societal risk governance) of Section 3.5, relies on journals focusing on ‘*Psychology, Education, Social*’ and ‘*Health, Nursing, Medicine*’ on the global science map of Section 3.4, and bridges the scientific domains ‘*#5 Psychology and Social Sciences*’ and ‘*#1 Biology and Medicine*’ on the global science map of Section 3.3. The highest cited references in this cluster are [16], which describes the evolution of 20 years of risk perception and risk communication research; [98], which addresses the issue of various scales of risk as a challenge for risk communication; and [99], which presents an analytical–deliberative process for risk communication.

## 4. Discussion

### 4.1. Interpretation of the Results

The analysis of research outputs in the risk communication research domain in Section 2.1 shows a fast accelerating growth, especially over the last two decades. While it is tempting to conclude that risk communication has become a more popular research topic, this development should be understood in light of general trends in academic publishing. It has been shown that publication rates have increased sharply across the entire scientific enterprise [100], and similar surges in publication rates have been observed in other risk-related scientometric analyses [33,35,41]. Hence, it is not entirely clear whether the increased risk communication research outputs are indeed indicative of its relative increased academic significance, whether these are the result of the increased relevance in societal governance or medical contexts, or whether the trends are caused by internal dynamics of academia.

Focusing on the geographical distribution of the research outputs shown in Section 3.2, it is very clear that, for the time being, the domain is very strongly dominated by research originating from Western countries, with the United States of America being by far the largest contributor. Only some Western European countries also have significantly contributed to the domain, with other geographical regions displaying a much lower research output. The research impact shows a similar picture. There are, however, some signs that risk communication research is also gaining importance in non-Western countries, with research originating from, e.g., China, South Korea, and Brazil, being relatively sizeable, especially more recently. This may indicate that the Western dominance may decrease in the future and that new perspectives may enter the research domain.

The relative dearth of risk communication research in South American, Eastern European, African, Middle Eastern, Asian, and Oceanian countries/regions may be a reflection of the governance structures of those societies, because governance approaches and mechanisms are necessarily embedded in cultural, organizational, and political contexts [11]. In this context, it is noteworthy that risk communication processes, especially when related to societal governance, have been strongly linked to deliberative processes and hence often assume certain forms of democratic societies with a large role for public engagement and participation [101]. Nevertheless, informing the public in disaster or crisis situations, e.g., related to public health or natural hazards, is likely important irrespective of the political governance style.

The activity in the broad co-citation clusters of Section 3.6 and the research activity across scientific categories of Section 3.3 indicates that risk communication research follows general societal concerns: while health, medicine, and environmental issues are of continuous relevance, concerns about specific technologies such as nuclear power are more transitory. The recent focus on meteorology and atmospheric sciences and geosciences furthermore indicate a broad trend in increased focus on effects of climate change and natural disasters. These broad trends are also found in risk perception research [102], which is closely linked with risk communication as these are commonly discussed together, especially in a societal risk governance context [11,97].

The scientific category analysis of Section 3.3 and the journals’ distribution and intellectual basis analysis of Section 3.4 show that the risk communication body of knowledge is interdisciplinary. Typically, research relies on social science knowledge as a base field, which is linked to more domain-specific knowledge related to medical, environmental, technical, or physical hazards. The results of the co-citation analysis and research fronts described in Section 3.6 show that applied research in risk communication appears to follow large societal trends and topics of societal concern, including public health, nuclear power, epidemics, natural disasters, and food regulation.

The analysis of narrative patterns in Section 3.5 shows that there are two major clusters in terms: one related to societal risk governance and one addressing medical risk communication. These address largely different problems, in that the former focuses on communication between different societal stakeholders with possibly conflicting value systems and objectives, while the latter is concerned with interpersonal communication in a trust relationship between medical practitioner and patient (or family). The term analysis of Figure 6, Figure 7 and Figure 8 and Table 7 show that these clusters are mostly disjoint. The results of the co-citation cluster results of Section 3.6 also show that medicine-related risk communication knowledge remains mostly in that broad subdomain of the research field, while application-specific knowledge related to other societal risks also is rather contained, as can be judged from the mostly very high silhouette values.

Finally, in the analysis of journal distribution in Section 3.4, it is noteworthy that *Risk Analysis* and *Journal of Risk Research*, which have been identified as core journals in risk and safety research [103], concentrate a large body of work on risk communication, while also having a significant scientific impact in this domain. This can be seen to support arguments that risk research is not merely an interdisciplinary or transdisciplinary research activity, but that it has its own foundational basis of concepts, theories, models, and approaches, and that it hence could be seen as a research domain in its own right [67].

As noted by one of the reviewers of an earlier version of this manuscript, the answers found to the questions stated in the introduction are in line with what knowledgeable domain experts would expect. Through a data-driven approach, the results obtained in this work substantiate these intuitive expert insights, providing evidence for the stated research questions, while raising new questions and research directions, as outlined next.

### 4.2. Future Work

Based on the results of Section 3 as discussed in Section 4.1, a number of avenues for future work can be identified, either to further develop the research domain of risk communication itself, or to better understand how it has developed as a domain of scientific activity.

Considering that risks are mediated and socially conveyed differently across varying cultural traditions [104], the lack of risk communication research in non-Western societies may result in a culturally biased approach. Hence, available theories, models, or conceptual frameworks for risk communication may need modification or elaboration to account for different social traditions, world views, or knowledge systems. More future research in non-Western countries may therefore lead to new fundamental insights and applications in the research domain.

Based on the findings that applied risk communication research focuses on issues of contemporary societal importance, there are various new directions for future risk communication research. Major global risks are one important line of work. Judging from The Global Risks Report 2020 [105] and considering that perceptions of importance of societal risks are usually mostly driven by the severity of potential impacts [106], such research could focus on climate action failure, weapons of mass destruction, biodiversity loss, extreme weather, water crises, information infrastructure breakdown, natural disasters, cyberattacks, human-made environmental disasters, and infectious diseases. Other topics for risk communication may concern new technological developments or consumer products. Risk communication research may be especially relevant where such technologies lead to concerns about human health or safety, in particular in contexts where uncertainty and ambiguity are societally important dimensions of risk. One example of this is the human health concerns related to the adoption of 5G wireless communication technology [107], about which conspiracy theories circulate on social media platforms, linking 5G technology to the COVID-19 pandemic [108]. Another example may be the industrial developments towards autonomous cars and maritime autonomous surface ships. Risk perceptions may be important factors in certain consumer or societal stakeholder groups to oppose the adoption of such new technologies [15,109]. Risk communication research can help to inform and interact with the public for such new developments.

Based on the finding that risk communication research related to societal risk governance issues and research in medical contexts are as yet largely separated areas of work, it may be fruitful for future research to identify links between certain themes within these subdomains. Such knowledge exchange can, for instance, lead to new conceptual, theoretical, or methodological approaches.

While scientometrics analyses are well suited to obtain high-level insights into a research domain in terms of its structure, patterns, and evolution, the existing scientometric techniques are ill-suited to detect research gaps, recent patterns, or research directions related to specific frameworks or approaches. Other review types, such as critical reviews, meta-analyses, or systematic reviews, are much more amenable to these goals (see [29]). Several such narrative reviews have already been published. Hence, the current work should be seen as complementary to those, aiming to achieve different aims.

Finally, further work can be directed to better understanding the development of the risk communication research domain. Example research questions in this line of work can include, for instance, how risk communication has impacted the disciplines it is associated with; what relationships between risk communication and anthropology exist and how knowledge of the latter may be used to advance the former especially when dealing with non-Western societies; to what extent and how risk communication research has influenced political science research and actual political decision making processes; and to what extent risk communication research has helped to expand risk research or establish it as an independent domain of scientific activity. Narrative reviews and other research methods are better suited for such more in-depth questions about the risk communication research domain than the scientometric analyses presented here. Nevertheless, the results of Section 3 may serve as a basis for directing such future research.

### 4.3. Limitations

As in any study, it is important to be aware of the limitations of the presented work. First, the analysis is conditional to the search strategy described in Section 2.1. While a title-based search strategy is widely applied in broad research domains to ensure relevance of the identified documents, using other search terms; searching in title, abstract, and keywords; or using a database other than WOSCC may affect the articles which are found and hence the results. Finally, the language restriction to English language publications may also induce some blind spots in the analysis.

It is noted here that using other terms such as ‘crisis communication’, ‘emergency communication’, or ‘disaster communication’ will almost certainly lead to detecting other patterns and trends. While these themes clearly have a close relationship to risk communication, as evidenced, e.g., by the results of the terms analysis (Section 3.5) or the co-citation clusters (Section 3.6), the choice has been made in this article to restrict the search to ‘risk communication’. This is done for two reasons. First, including other terms blurs the scope of the domain of research which is intended to be covered, as it opens questions as to why then for instance ‘hazard communication’ or ‘safety communication’ are not included. The authors believe that a clearly delineated focus on risk communication is preferable from a conceptual and methodological point of view. The second reason relates to the meaning of the risk concept as it contrasts to other related terms such as those mentioned above. While there is no scientific agreement about what exactly ‘risk’ means, it is broadly agreed that this carries the notion of possible or uncertain future events [110]. Thus, it may be deduced that risk communication focuses primarily on informing and interacting about events which have not yet happened. In contrast, crisis, emergency, or disaster communication is more focused on providing information about events which have already occurred or are ongoing [111]. While this delineation is not exact due to linguistic ambiguities, risk communication for example relates more to stakeholder processes in preparedness planning for natural or technological disasters, while crisis communication would focus on what information to provide, when, and how, to affected people in an ongoing disaster. Follow-up research may explore the domains of research covered by the other mentioned search terms, from which more conclusive statements about their specific relationships can be made.

In the analyses involving temporal evolutions of geographical research productivity (Section 3.2), active scientific categories (Section 3.3), narrative patterns (Section 3.5), and co-citation clusters (Section 3.6), the average publication year is used as a metric. While averages may hide significant information about the shape of the distribution, e.g., its variance or skewness, averages are commonly used in scientometric research to obtain high-level insights into the development of a research domain [28].

A common criticism of scientometric analyses is the use of citation metrics such as total number of citations for determining impact and detecting patterns. As citations need time to accumulate, the reliance on number of citations may cause some important trends to be missed, especially in more recent research. This can be seen, e.g., in Table 4, where some scientific categories with more recent average years of publication do not have very high average citation scores. This may be because of the actual lower impact in the academic community, but the measures are also confounded by the shorter time of citations to accumulate to this research. Furthermore, using citations as a proxy for significance of research contributions is controversial [112,113], e.g., because of the potential for manipulation and lack of consideration of why an article was cited. Consequently, the presented scientometric analysis should not be seen as an endorsement of the correctness, value, or effectiveness of the highlighted risk communication research works. Instead, the analyses should rather be understood as descriptive of the development of the field and the countries, scientific categories, journals, terms, and references which have jointly forged and shaped the research domain to what it currently is.

## 5. Conclusions

In this work, a scientometric analysis of the risk communication research literature has been presented, spanning the period from 1985 to 2019. Various scientometric methods and visualization tools are applied to determine temporal trends and geographical patterns, contributing scientific categories and domains, the distribution of contributing journals and the intellectual basis of the domain, narrative patterns, and the evolution of the research domain using co-citation clusters. The analyses provide unprecedented insights into the structure, patterns, and developments of the risk communication domain, leading to several avenues of future research. The following main key conclusions are drawn:

(i) Risk communication research has grown exponentially, especially with a very significant increase since the early 2000s.
(ii) The domain is dominated by Western science, primarily from the USA and Western European countries, with non-Western research however recently emerging.
(iii) The research domain is highly interdisciplinary, where typically knowledge from *‘Psychology and Social Sciences’* is combined with application-specific knowledge, with the domains ‘*Biology and Medicine*’, and ‘*Ecology and Environmental Science and Technology*’ being the most prominent.
(iv) The most important journals in the field are *Risk Analysis* and *Journal of Risk Research*, which may suggest that risk research can been seen as a scientific domain in its own right.
(v) There are two main, largely disconnected, narrative clusters in risk communication research. The first cluster is that of communication between medical practitioners and patients, and the other cluster concerns stakeholder communication for societal risk governance. The dominant narratives in the former concern interventions, decision making, and various medical conditions, whereas the latter focuses on societal risks such as public health, food, floods, or disease outbreaks.
(vi) Risk communication research originates from a practice-oriented need to communicate regarding industrial (environmental) contamination and public health. Most subsequent research clusters address particular medical issues or societal concerns, including nuclear power, epidemics, or natural disasters. Apart from such application-oriented work, there are also some clusters that address risk communication models or theories or that study risk communication effectiveness. Many clusters are quite disjoint. This indicates that knowledge exchange between application domains is not very significant, which may therefore be a fruitful direction for future scholarship.

Apart from providing insights into the structure and evolution of the research domain and leading to the formulation of several avenues for future research, the results are considered particularly useful for early career researchers who are relatively new to the very extensive domain of risk communication research. They can also assist experienced academics in navigating the fast-increasing volume of research, either for teaching or research purposes. The results can also be useful for journal editors, to position their journal in the wider body of scientific work or to identify hot topics, for instance, for deciding on opening a special journal issue on a certain topic.

## Figures and Tables

**Table 1:** Descriptive information of the dataset on risk communication research.

Description	Results	Description	Results
Period	1985–2019	Authors	3137
Sources (journals, books, etc.)	523	Author appearances	3944
Documents	1196	Authors of single-authored documents	296
Journal articles	715	Authors of multi-authored documents	2841
Conference articles	247	Single-authored documents	358
Review articles	58	Avg. number of documents per author	0.381
Editorials	111	Avg. number of authors per document	2.62
Other (letter, note, etc.)	65	Avg. number of co-authors per document	3.3
Author’s keywords	1668	Collaboration index	3.39
Avg. citations per document	14.82		

**Table 2:** Overview of scientometric techniques and tools used to answer the research questions.

ID	Research Question Focus	Scientometric Technique and Tools	Reference
RQ1	Publication output trends	Regression analysis	-
		Publication analysis (Bibliometrix)	[42]
RQ2	Geographic patterns	Publication analysis (Bibliometrix)	[42]
		Visualization of similarities (VOSviewer)	[45]
RQ3	Scientific categories	Visualization of similarities (VOSviewer)	[45]
		Global science map overlay	[46]
RQ4	Journal knowledge flow	Journal distribution analysis (CiteSpace)	[47]
		Journal-based dual-map overlay	[48]
RQ5	Narrative topics	Automatic term identification method	[49]
		Visualization of similarities (VOSviewer)	[45]
RQ6	Research clusters and fronts	Co-citation analysis (CiteSpace)	[47]

**Table 3:** High-productivity countries/regions in risk communication.

Countries/Regions	NP	TC	AC	APY	Degree
United States of America	502	9851	19.62	2008.76	22
United Kingdom	177	3147	17.78	2010.55	22
Germany	93	920	9.89	2011.83	14
Netherlands	68	1136	16.71	2012.28	15
Canada	58	1019	17.57	2010.98	12
Australia	45	572	12.71	2012.96	9
Japan	35	204	5.83	2012.17	6
Italy	29	200	6.90	2013.28	6
Sweden	28	184	6.57	2011.96	8
Switzerland	23	477	20.74	2013.04	9
People’s Republic of China	20	109	5.45	2015.65	6
France	20	142	7.10	2011.80	10
Norway	16	94	5.88	2011.38	7
South Korea	14	49	3.50	2017.00	2
Spain	13	77	5.92	2011.38	5
Brazil	12	77	6.42	2013.33	4
Belgium	10	150	15.00	2009.70	3
Denmark	10	95	9.50	2011.50	6
Singapore	9	39	4.33	2012.67	5
Thailand	8	48	6.00	2012.63	3
Israel	6	91	15.17	2013.83	5
Mexico	6	62	10.33	2010.17	3
New Zealand	6	61	10.17	2011.83	1
Portugal	6	4	0.67	2013.83	5

Note: NP = number of publications; TC = total citations; AC = average citations per paper; APY = average publication year; Degree = number of collaborating countries/regions of a node in the network. Colors in columns NP, TC, and AC indicate the relative impact of the countries, with deeper shades of red signifying more impact and deeper shades of blue less impact. The color scheme in the column ‘Degree’ is similar, with deeper red shades signifying more international collaborations, and blue shades fewer collaborations. In the column APY, deeper shades of red signify more recent research contributions, while deeper blue shades indicate more temporally distant publications.

**Table 4:** Scientific categories with more than 20 papers on risk communication published.

Scientific Category	NP	APY	AC	Cluster
Public, Environmental, and Occupational Health	362	2007.47	17.17	5
Environmental Sciences	105	2007.97	8.63	3
Social Sciences, Mathematical Methods	89	2000.45	36.45	5
Mathematics, Interdisciplinary Applications	88	2000.57	36.70	4
Social Sciences, Interdisciplinary	86	2011.35	13.86	5
Communication	67	2009.42	24.10	5
Medicine, General and Internal	67	2008.88	25.24	1
Toxicology	65	2009.88	7.42	1
Pharmacology and Pharmacy	64	2011.78	2.77	1
Oncology	42	2011.14	11.98	1
Psychology, Multidisciplinary	42	2010.07	8.48	5
Health Care Sciences and Services	42	2009.21	32.74	5
Water Resources	41	2010.78	7.98	3
Environmental Studies	39	2010.74	8.95	5
Food Science and Technology	39	2010.77	5.79	1
Meteorology and Atmospheric Sciences	34	2012.97	12.35	3
Health Policy and Services	30	2010.07	35.70	5
Information Science and Library Science	30	2009.20	38.17	5
Radiology, Nuclear Medicine, and Medical Imaging	29	2009.90	6.38	1
Nuclear Science and Technology	28	2007.75	6.54	2
Geosciences, Multidisciplinary	24	2014.79	15.00	3
Social Sciences, Biomedical	24	2010.25	20.00	5
Medical Informatics	23	2009.61	52.13	1
Engineering, Civil	21	2005.00	2.52	4

Note: NP = number of publications; APY = average publication year; AC = average citations per paper; Cluster = main science domain as per the global science map of Figure 4. Colors in columns NP and AC indicate the relative impact of the scientific categories, with deeper shades of red signifying more impact and deeper shades of blue less impact. In the column APY, deeper shades of red signify more recent research contributions, while deeper blue shades indicate more temporally distant publications. The colors in the column ‘Cluster’ correspond to the colors of the clusters as shown in Figure 4.

**Table 5:** Top 10 highly productive journals and highly cited journals in risk communication research.

No.	Citing Journals	NP	Cited Journals	NC
1	*Risk Analysis*	87	*Risk Analysis*	1705
2	*Journal of Risk Research*	61	*British Medical Journal*	459
3	*Drug Safety*	24	*Medical Decision Making*	423
4	*Journal of Health Communication*	22	*Journal of Risk Research*	391
5	*Pharmacoepidemiology and Drug Safety*	19	*Science*	372
6	*Health Physics*	18	*Patient Education and Counseling*	278
7	*Annals of Behavioral Medicine*	13	*Journal of Health Communication*	266
8	*Human & Experimental Toxicology*	11	*Journal of the American Medical Association*	244
9	*Medical Decision Making*	11	*Health Psychology*	201
10	*Patient Education and Counseling*	11	*New England Journal of Medicine*	179

Note: NP = Number of publications; NC = Number of citations.

**Table 6:** Citation trends of risk communication at a domain level.

No.	Citing Domain	Cited Domain	Z-Score
1	Medicine, Medical, Clinical	Health, Nursing, Medicine	5.690
2	Psychology, Education, Health	Health, Nursing, Medicine	4.441
3	Psychology, Education, Health	Psychology, Education, Social	6.304

**Table 7:** List of risk communication terms with more than 20 term occurrences.

Cluster A	NOC	APY	AC	Cluster B	NOC	APY	AC
Agency	75	2009.84	9.80	Patient	160	2011.20	19.99
Government	69	2010.35	12.45	Intervention	83	2011.10	23.75
Stakeholder	63	2012.89	8.78	Decision Making	78	2009.79	36.81
Organization	61	2011.39	10.74	Probability	57	2008.93	27.88
Case Study	52	2009.60	14.52	Woman	57	2009.68	17.81
Industry	47	2009.66	14.11	Age	55	2011.33	23.76
Crisis	45	2011.07	17.02	Cancer	53	2010.94	21.26
Consumer	44	2011.30	15.43	Condition	52	2009.85	18.96
Public Health	42	2010.62	10.52	Test	44	2009.82	32.70
Food	40	2010.28	10.03	Treatment	42	2010.95	23.05
Authority	37	2011.32	12.43	Frequency	41	2011.90	34.93
Product	36	2012.56	10.53	Family	39	2010.79	18.05
Audience	35	2009.66	22.46	Preference	38	2009.68	22.50
Communicator	35	2009.80	29.49	Trial	38	2011.32	20.66
Scientist	35	2009.57	12.17	Intention	37	2013.35	25.00
Flood	33	2013.52	17.15	Child	36	2011.58	10.64
Failure	32	2009.09	17.50	Skill	36	2007.28	50.64
Disaster	31	2013.00	17.84	Physician	35	2008.91	21.69
Debate	30	2008.80	17.60	Percentage	33	2010.18	24.58
Citizen	29	2008.55	9.45	Care	30	2010.83	22.23
Outbreak	29	2013.28	13.00	Clinician	30	2010.77	34.57
Public Perception	29	2007.00	21.17	Medicine	29	2011.28	14.66
Regulator	29	2010.48	13.45	RC Tool	29	2011.76	13.07
Communication Effort	28	2009.68	23.14	Consultation	28	2008.29	27.82
Contamination	28	2011.79	6.29	Scenario	28	2010.32	21.21
Efficacy	28	2012.14	23.43	Training	28	2009.61	21.54
Emergency	28	2013.64	16.96	Score	27	2011.78	33.70
Campaign	27	2013.15	13.56	High Risk	26	2011.69	24.73
Dialogue	27	2006.85	15.78	Relative Risk	26	2010.88	45.23
Europe	27	2011.93	12.07	Risk Factor	26	2012.46	17.54
Food Safety	25	2011.40	11.08	Scale	26	2010.15	46.15
Preparedness	25	2014.32	13.20	Anxiety	25	2012.92	20.76
Requirement	25	2009.88	16.60	Programme	24	2011.25	20.46
Chemical	23	2006.43	13.65	Provider	24	2012.33	11.13
Social Medium	23	2016.13	6.57	Subject	24	2007.29	37.29
Climate Change	22	2015.09	10.77	Breast Cancer	23	2012.39	14.74
Credibility	22	2007.95	33.41	Comprehension	23	2010.91	21.30
City	21	2011.38	25.81	Parent	23	2011.52	21.74
Identification	21	2013.19	8.38	Participation	23	2009.26	26.04
Public Participation	21	2003.76	26.19	Majority	22	2009.32	20.59
				Systematic Review	22	2013.50	38.14
				Qualitative Study	21	2011.57	21.05
				Risk Reduction	21	2011.95	17.48

Note: NOC = number of occurrences; APY = average publication year; AC = average number of citations. Colors in columns NOC and AC indicate the relative impact of the terms, with deeper shades of red signifying more impact and deeper shades of blue less impact. In the column APY, deeper shades of red signify more recent research contributions, while deeper blue shades indicate more temporally distant publications.

**Table 8:** Research clusters in risk communication research in connected largest network, clusters including a minimum of five references.

ID	Cluster Name	Size	Avg (YR)	Silhouette	LLR Title Terms	Research Front
α	Pictographs	84	2007	0.769	decision outcome; presenting quantitative information; patient decision aid developer; pictographs; numeracy	[60]
β	Learning through conflict	78	1989	0.931	risk communication challenge; realistic strategy; correcting mental model; learning through conflict	[61]
γ	Food risk communication	75	2003	0.867	food risk communication; rational choice regulation; uncertainty transfer	[62]
δ	Rational public discourse	69	1988	0.898	rational discourse; risk communication effectiveness; aspen-EPA superfund controversy; rhetorical stases	[63]
ε	General practice patient involvement	61	2001	0.835	making skill development; risk communication aid; shared decision; general practice	[64]
ζ	Flood risk communication	56	2009	0.852	flood risk communication; prevention-focused motivation; linking social capacities; NRC report; Roger Kasperson	[65]
η	Epidemic and bioterrorism	55	1996	0.934	urban setting; communication challenge; Nile virus epidemic	[66]

Note: Size = number of publications in the cluster; Avg (YR) = the average publication year of the references in the cluster; LLR Title Terms = terms in the title based on the log-likelihood ratio; Research Front = article which cited most papers from the cluster.

**Table 9:** Top five highly cited references with at least five citations received in the largest co-citation clusters (NP > 50) of Figure 10, see also Table 8.

ID	Citations	First Author	PY	Source (Journal Article or Book)	Reference
α	20	Lipkus, I.M.	2007	*Medical Decision Making*	[68]
α	17	Paling, J.	2003	*British Medical Journal*	[58]
α	10	Gigerenzer, G.	2003	*British Medical Journal*	[69]
α	10	Peters, E.	2006	*Psychological Science*	[70]
α	10	Visschers, V.H.M.	2009	*Risk Analysis*	[21]
α	10	Galesic, M.	2009	*Health Psychology Journal*	[71]
β	17	National Research Council (US)	1989	*Improving Risk Communication*	[72]
β	8	Covello, V.T.	1988	*Risk Communication, Risk Statistics, and Risk Comparison*	[73]
β	5	Bostrom, A.	1992	*Journal of Social Issues*	[74]
β	5	Kraus, N.	1992	*Risk Analysis*	[75]
γ	14	Slovic, P.	2000	*The Perception of Risk*	[76]
γ	13	Pidgeon, N.	2003	*The Social Amplification of Risk*	[77]
γ	13	Morgan, M.G.	2002	*Risk Communication: A Mental Models Approach*	[78]
γ	11	McComas, K.A.	2006	*Journal of Health Communication*	[18]
γ	10	Lofstedt, R.E.	2005	*Risk Management in Post-trust Societies*	[79]
δ	8	Krimsky, S.	1988	*Environmental Hazards: Communicating Risks*	[80]
δ	7	Covello, V.T.	1988	*Seven Cardinal Rules of Risk Communication*	[81]
δ	5	Kasperson, R.E.	1988	*Risk Analysis*	[82]
δ	5	Sandman, P.M.	1987	*Journal of Communication*	[83]
ε	16	Edwards, A.	2002	*British Medical Journal*	[84]
ε	11	Edwards, A.	2000	*Medical Decision Making*	[85]
ε	8	Lipkus, I.M.	1999	*Journal of the National Cancer Institute Monographs*	[86]
ε	6	Rothman, Alexander J.	1999	*Journal of the National Cancer Institute Monographs*	[87]
ε	5	Edwards, A.	1999	*Medical Decision Making*	[20]
ε	5	Stone, E.R.	2003	*Journal of Organizational Behavior and Human Decision Processes*	[88]
ε	5	Slovic, P.	2000	*Law and Human Behavior*	[89]
ε	5	Hallowell, N.	1997	*Journal of Genetic Counseling*	[90]
ε	5	Guadagnoli, E.	1998	*Social Science & Medicine*	[91]
ε	5	Towle, A.	1999	*British Medical Journal*	[92]
ζ	15	Kasperson, R.	2014	*Journal of Risk Research*	[93]
ζ	14	Kellens, W.	2013	*Risk Analysis*	[94]
ζ	13	Wachinger, G.	2013	*Risk Analysis*	[95]
ζ	11	Bubeck, P.	2012	*Risk Analysis*	[96]
ζ	9	Renn, O.	2008	*Risk Governance: Coping with Uncertainty in a Complex World*	[97]
η	8	Fischhoff, B.	1995	*Risk Analysis*	[16]
η	7	Calman, K.C.	1997	*British Medical Journal*	[98]
η	5	Renn, O.	1999	*Environmental Science & Technology*	[99]

Note: PY = publication year.
